# Supplementary material for: Patterns of Intron Gain and Loss in Fungi
Source: PLoS Biol. 2004 Nov 30;2(12):e422. doi: 10.1371/journal.pbio.0020422 (PMC532390; doi:10.1371/journal.pbio.0020422)
Supplement: Table S1 — Also available at http://genes.mit.edu/NielsenEtAl/. (4.3 MB ZIP). [file pbio.0020422.st001.zip › NielsenEtAl/html/343.html]

AN2946.1.NCU02515.1.MG07745.1.FG05621.1


```
 CLUSTAL W (1.82) Multiple Sequence Alignments - Introns Inserted


Sequence 1: NCU02515.1	895 aa
Sequence 2: MG07745.1	938 aa
Sequence 3: FG05621.1	911 aa
Sequence 4: AN2946.1	880 aa
Alignment Length: 955 aa
Number Identitical Residues: 354 aa
Alignment Score (without introns) 19599


MG07745.1 	MPVDKKSGNGRPS---LDSVSSISTTSIVFDHLNDLEIHKKHRPHLMTESIRRDDHLSDF
NCU02515.1	-----------MS---QETDSTVSTTSIVFDRIQERLDTKEFP--------ARG---TDG
FG05621.1 	MALDMSTSERRYSDDSDDSISTASTTSFVFERIQEKTEMDAGK---------DNNDPRAL
AN2946.1  	----MRSSEDREDSELLPANRPRSPSRSSYDSDDSGLSVDSILE--------EQKYNAAT
          	      :..   ...   :  . *.:   ::  :.    .              .     

MG07745.1 	DDDDPLKQEMDGEGDLETGPFLAGR------DAYTFGGGKYMDRKFRRIMIVVAGLLVAA
NCU02515.1	DDNDSLKDELNND-DLETGPFLGNASPSSRSNQRSSADGQRMDRSLRRWLFIVSGALVAT
FG05621.1 	DDDDPLKEE----DDLETGPFLGPG---------ATLHRQPMDKKLRRILIIAGAAFVAL
AN2946.1  	NETLGLPQEMR-YHDEEGGEAGSNE----------ALHTKASSSRSRRLLWLVVLLCCGG
          	::   * :*     * * *   .                :  .   ** : :.     . 

MG07745.1 	WVGGLFVYIAS--KSYKHAT-TVEHDPEKTKAAALTSGKKITMDQVRSGTWYAASHDITW
NCU02515.1	WVIGLIFFVSS--KAYKPSS-SFAHDPQAT--VTHGSGKKVTLDQVLNNEWRAKSHSISW
FG05621.1 	WLAGLGIFLYK--GSYHHES-DTEHDPDAA---TRGSGKTVTMDQLFDGSWQAKSHSISW
AN2946.1  	WVVAFVLFITQGRADYRTATDELQSDNSGSFSDGTSSGKPLTLQQVLSGVFLPRGHAISW
          	*: .: .:: ..   *:  :.    * . : :    *** :*::*: .. : . .* *:*

MG07745.1 	IAGPKGEDGLMIEVGADGKD-YIVIEDGRAENPDSSAE-AEVDVTASRRTLMKNGSFRYQ
NCU02515.1	IAGVNGEDGLLLEKEGANKD-YLVVEDVRAQNPSS------VEASKSK-TLIKDKLFEFA
FG05621.1 	IAGPDGEDGLLLEVGASEKP-YIVVEDIRKDKENRAPINTEIKASKSR-TLMKDNFFVHD
AN2946.1  	VAGPDGEDGLLIERGEDDEAGYLRINDIRQD-------------GKVNRVLMQKPTVGVD
          	:** .*****::*     : .*: ::* * :                . .*::.  .   

MG07745.1 	GRMHIPSGAEPSRDQQKVLLRTEQKSNWRHSHTACYWIWDVATETAEPLIPASPDARVQN
NCU02515.1	NKTYWPTVTVPSRDLKKVLLATDVQNNWRHSYYAVYWIFDVETQQAEPLVPYDADARLQL
FG05621.1 	GNQYTPDWNEPSPDLKKVLLGVNKKKNWRHSFSAIYFILDVETQQAEPLVPGKPDARIQL
AN2946.1  	GRTIKPSATRPSPDLKKVLIISNQEKNWRHSFTASYWIFDVETQTAEPLDPNNIDGRVQL
          	..   *    ** * :***:  : :.*****. * *:* ** *: **** * . *.*:* 

MG07745.1 	AQWSPTSDAIVFTRENNLYLRTIGSKK-VTQITKDGGADLFYGVPDWVYEEEVIAGASTT
NCU02515.1	ASWSPTSDAIVYTRDNNMFLRKLDSDK-IVQITRDGSADVFNGVPDWVYEEEVLASGVAT
FG05621.1 	ATWSPKSDAVSYTQDNNLYIRRLDDKKNVVQITKDGGPEYFYGIPDWVYEEEVFSGRSAT
AN2946.1  	ALWSPKSDAIAFVRDNNLYLRKLSSER-VVPITKDGGEQLFYGVPDWVYEEEVFSGNSVT
          	* ***.***: :.::**:::* :...: :. **:**. : * *:*********::.  .*

MG07745.1 	WWSEDGKYVAFLRTNETGVPAFPVQYFMSRPSGASPKAGEETYPEVRDIKYPRSGSHNPV
NCU02515.1	WWSEDGNYVAFLRTNETGVPEYPIQYFVSRPSGEEPKPGEENYPEVRQIKYPKAGAHNPI
FG05621.1 	WWSEDGKYLSFLRTNETGVPEFPVQFFIKRPSGTDPEKGEEAYPEVEQIKYPKAGAHNPV
AN2946.1  	WWSEDGSQIAFIRTNESAVPEFPVQYFLSRPSGKKPQPGLENYPEVREIKYPKAGAPNPF
          	******. ::*:****:.** :*:*:*:.**** .*: * * ****.:****::*: **.

MG07745.1 	VDVQFYDIARGDVFTVDINGGFADEDRLITTVLWAGS-QVIVKETNRVSDIMRVVLIDVK
NCU02515.1	VDLKFYDVKRGDVFSVDISGRFADDDRLITEVIWAGK-QVLIKETNRVSDVMRVVLVDVG
FG05621.1 	VDLLFYDIAKKDVFSVDIDGAFADDDRIINNLLWAGD-KAVVKETNRVSDILKVVLIDVT
AN2946.1  	VNLQFYDVEQGEVFSVDTPDDFDDDDRLIIEVIWAAKGKVLVRTTNRESDILKVFLVDTE
          	*:: ***: : :**:**  . * *:**:*  ::**...:.::: *** **:::*.*:*. 

MG07745.1 	KRTGKTTRTVDVGKIDGGWFEISQNTKYIPADPKKGRDQDGYIDTVIHNDGDHLAYFSPP
NCU02515.1	SRTGKAVRTVDVNDIDGGWFEISHKTKFIPADPANGRPDDGYVDTIIHNNGDHLAYFTPL
FG05621.1 	SRKGKTVNTVDVDKIDGGWFEISHKTAYIPADPANGRDHDGYVDSYLHEGYEHLAYFTPL
AN2946.1  	SRESKLIRIQDISELDGGWVEPTQSVRFIPADPDKGRPFDGYLDTVVHEGYDHLAYFTPL
          	.* .*  .  *:..:****.* ::.. :***** :**  ***:*: :*:. :*****:* 

MG07745.1 	DNPDPIMLTSGPGWEVVDAPSAVDLENNLVYFIATKEGSTQRHVYSVQLDGKNMKSFTDT
NCU02515.1	DNPNPIMLTSG-DYEVVDAPSAVDLQRNLVYFVSTKESSIQRHVYQVKLTGEDMTPVTDT
FG05621.1 	DNPDPIMLTKG-EWEIDDAPSAIDLANNLVYFIAAKESSIQRHVYSVKLDGTKLEALTDP
AN2946.1  	DNPEPIMLTSG-EWEVVDAPTAVDLTRGLVYFIATKEAPTERHLYRVRLDGSDLTPLTDT
          	***:*****.*  :*: ***:*:** ..****:::**.. :**:* *:* * .: ..**.

MG07745.1 	EAKGYYDVSFSSGAGYALLSYKGPKIPWQKVVSTPANNQRYEVLIEENKELAESARKYDL
NCU02515.1	SKEGYYAISFSTGAGYALVSYQGPNIPWQKVISTPSNPDKYEHVVEENKDLAEAAKKHEL
FG05621.1 	ETEAYYDASFSKSAGFVLLSYRGPKVPSQKVISTPSSVHDYEHVIEDNAELAERARSHEL
AN2946.1  	SQPGYYSVSFSDGAGYALLSYQGPSIPWQSIISTEGEKTTTLRIIEDNTDLSKLVAQYAL
          	.  .**  *** .**:.*:**:**.:* *.::** ..      ::*:* :*:: . .: *

MG07745.1 	PLLNYGTLNV-EGVELNYLERRPPQFSEKKKYPVLFHQYSGPGSQSVSQKFSVDFQSYVA
NCU02515.1	PINIYGTINV-DGVELNYIERRPPHFDKNKKYPVLFQQYSGPVSQTVKKTFAVDFQSFVA
FG05621.1 	PVMKYGTLDLGDGIVVNYVERRPPHFDKKKKYPVLFQQYSGPGSQSVTKRFAVDFQAYVA
AN2946.1  	PTENYQNITI-DGFTLQVVERRPPHFNPARKYPVLFHLYGGPGSQTVDRRFNVDFQSYVA
          	*   * .: : :*. :: :*****:*.  :******: *.** **:* : * ****::**

MG07745.1 	ASLGYIVVTFDGRGTGFIGRKNRVLVRSRLGEIEAQDQIAAAKHWASLGYVDPSRIAIWG
NCU02515.1	AGLGYICVTVDGRGTGFIGRKNRVIIRGNLGTWESHDQIAAAKHWAQKDYIDEDRLAIWG
FG05621.1 	SALGYLVVTVDPRGTGFLGRKHRVVVRSQLGVLESQDHIAAAQSFASRPYVDAERLAIWG
AN2946.1  	ASLGYIVVTVDGRGTGFIGRAARCIIRGNIGHYEAIDQIATAKNWAQKPYVDESRMAIWG
          	:.***: **.* *****:**  * ::*..:*  *: *:**:*: :*.  *:* .*:****

MG07745.1 	WSYGGFQTLKTLEADAGRTFSYGMAVAPVTDWRFYD~SIYTERYMLTPQQNEDGYTKSAV
NCU02515.1	WSYGGYMTLKTLEQDAGQTFKYGMAVAPVTDWRFYD~SIYTERYMRTPQTNPEGYESAAV
FG05621.1 	WSYGGFTTLKTLEQDAGRTFSYGMAVAPVTDWRFYD~SIYTERYMRTPQENPGGYDMSMI
AN2946.1  	WSYGGFMTLKTLEQDAGETFQYGMAVAPVTDWRFYD1SVYTERYMHTPQHNPTGYDNTSI
          	*****: ****** ***.**.*************** *:****** *** *  **  : :

MG07745.1 	HNVSALASNKRFLLMHGASDDNVHFQNSLTLLDKLDMGAVENYDVHVFPDSDHSIFFHNA
NCU02515.1	TNVTALSQNVRFLLMHGVADDNVHMQNSLTLLDALDQRSVENYDVQVFPDSDHGIYFHNA
FG05621.1 	ANATALGGNKRFLVMHGVADDNVHFQNSLTLLDSLDLAGVENYDVHVFPDSDHSIYFHNG
AN2946.1  	SDMAALHNNVRFLVIHGASDDNVHIQNTLTLIDKLDLASVQNYDVHFYPDSDHSIFFHNA
          	 : :**  * ***::**.:*****:**:***:* **  .*:****:.:*****.*:***.

MG07745.1 	NKIIYDK1LENWLVNAFNGEWLKVNNAKPVELVHPTQLVREREKAKLRKRWQEIQNK
NCU02515.1	NRIVFDK1LTNWLVNAFNGEWLKIANAQPNGMKR-----RALPTA------------
FG05621.1 	NRIVYDK1LRNWLINAFNGEWLKISDPKPHKEGV------EKEKRQLIADF------
AN2946.1  	HTMVYER1YLNVYMS-----------MKPDNL-------------------------
          	: :::::   *  :.            :*
```
